# Recent Advances in Immunotherapy for Melanoma: Perspectives on the Development of Novel Treatments: A Mini Review

**DOI:** 10.3390/cancers17132265

**Published:** 2025-07-07

**Authors:** Yusuke Muto, Taku Fujimura, Yoshihide Asano

**Affiliations:** Department of Dermatology, Tohoku University Graduate School of Medicine, Sendai 980-8574, Japan; yusuk0610@derma.med.tohoku.ac.jp

**Keywords:** melanoma, anti-PD-1 antibody, anti-CTLA-4 antibody, immunotherapy, IL-2, IFN

## Abstract

It has been more than a decade since anti-PD-1 and anti-CTLA-4 antibodies were first introduced for the treatment of unresectable melanoma. The advent of these immunotherapies has dramatically transformed the treatment landscape. In recent years, anti-PD-1 antibodies have become the cornerstone of melanoma therapy, and the development of new treatment regimens has advanced rapidly in both Eastern and Western countries. However, clinical practice has revealed lower response rates in East Asian melanoma patients compared to Caucasian populations. This discrepancy may be partially attributed to T cell immune exhaustion within the tumor microenvironment, although the detailed mechanisms remain unclear. Moreover, there is currently no established treatment for BRAF wild-type melanoma that is resistant to anti-PD-1 antibodies. This review discusses the currently available therapeutic strategies for advanced melanoma and addresses the aforementioned challenges, highlighting recent efforts in both Eastern and Western regions.

## Simple Summary

More than ten years have passed since anti-PD-1 and anti-CTLA-4 antibodies were first introduced as treatments for unresectable melanoma, marking a significant shift in therapeutic approaches. These immunotherapies have reshaped the management of the disease, with anti-PD-1 antibodies now forming the backbone of melanoma treatment. In both Western and Eastern countries, novel treatment regimens have progressed rapidly. Despite these advances, clinical outcomes in East Asian patients tend to be less favorable than those observed in Caucasian populations. This may be partly due to T cell exhaustion within the tumor microenvironment, though the precise underlying mechanisms remain to be elucidated. Furthermore, for patients with BRAF wild-type melanoma who have developed resistance to anti-PD-1 therapy, no standard treatment options currently exist. This review outlines current therapeutic strategies for advanced melanoma and explores ongoing efforts across different regions to overcome these challenges.

## 1. Introduction

In recent years, the introduction of immune checkpoint inhibitors has revolutionized melanoma treatment [1,2,3,4,5,6,7,8,9]. Among them, anti-PD-1 antibodies have emerged as a central therapeutic option, with novel treatment strategies progressing swiftly across both Western and Asian regions [6,7]. Historically, immunotherapies such as interferon, high-dose IL-2 therapy, and dendritic cell therapy have been attempted in the treatment of melanoma [1,2,3,4]. However, evidence-based therapeutic strategies had not been established until the advent of immune checkpoint inhibitors (ICIs) [5]. ICIs, such as anti-programmed death-1 (PD-1) and anti-cytotoxic T lymphocyte antigen-4 (CTLA-4) antibodies, are now standard treatments for advanced melanoma worldwide [6]. They have been shown to be particularly effective for non-acral melanomas that develop in sun-exposed areas, which are common in Caucasians [7]. On the other hand, ICIs are less effective for acral and mucosal melanoma subtypes, which arise in non-sun-exposed areas and are more prevalent in East Asian populations [8].

Since no effective treatments have yet been developed for BRAF wild-type melanoma resistant to ICIs [9], this review aims to explore alternative therapeutic options and evaluate potential treatment strategies beyond currently available ICIs.

## 2. Aim

We aim to discuss therapeutic alternatives to currently available ICIs and evaluate potential treatment strategies in this review.

## 3. Methods

### Search Strategy for the Literature Search

Areas covered: The literature review was performed on PubMed (1950~2025/4/30) to identify these drugs using search terms such as ‘melanoma’, ‘immunotherapy’, ‘immune checkpoint inhibitors’, ‘anti-PD-1 antibody’, ‘nivolumab’, ‘pembrolizumab’, ‘ipilimumab’, ‘relatlimab’, ‘anti-PD-1 antibody resistant’, and ‘clinical trial’. We summarized the results of literature search with relevant keywords in Table 1.

## 4. Previous Immunotherapy for Melanoma

### 4.1. Type I Interferon Therapy

Type I interferons (IFNs), including IFN-α, pegylated IFN-α, and IFN-β, are believed to enhance anti-tumor activity in melanoma through various immune mechanisms, such as increasing tumor-infiltrating immune cells, reducing circulating regulatory T cells (Tregs), and altering the STAT1/STAT3 signaling balance in tumor cells [10]. Additionally, type I IFNs are important for innate immune recognition of growing melanomas and promote the intra-tumoral accumulation of CD8α+ dendritic cells (DCs) in vivo [4]. IFN-α, a member of the type I IFN family, enhances the activity and cytotoxicity of natural killer (NK) cells and cytotoxic T lymphocytes (CTLs) [1,2].

In a randomized prospective phase III pilot trial, 61 patients with advanced melanoma were treated with dacarbazine (DTIC) alone or in combination with IFN-α. The median survival was 4.8 months (95% confidence interval [CI]: 2.0–8.0) in the combination group versus 7.2 months (95% CI: 4.4–9.0) in the DTIC-only group, showing no significant difference between the groups [1]. In 1995, high-dose IFN-α2b was approved by the U.S. Food and Drug Administration (FDA) as adjuvant therapy for high-risk melanoma [5]. Since higher proportions of CD8+ T cells and NK cells were observed in the peripheral blood of melanoma patients treated with IFN-α2b, it was expected to be a promising therapeutic option [3]. Although one study reported a statistically significant improvement in disease-free survival (DFS), a systematic review of randomized controlled trials found no clear benefit in overall survival (OS) for melanoma patients treated with IFN-α [6].

Overall, these IFN-based therapies did not demonstrate consistent clinical benefits in patients with advanced melanoma in past trials. In a multicenter phase II trial (DeCOG), 55 patients with stage IV melanoma were treated with a combination of sorafenib (a multi-tyrosine kinase inhibitor) and pegylated IFN-α2b [11]. Partial response (PR) was observed in two patients (3.6%), and stable disease was noted in fourteen patients (25.5%) at the 8-week evaluation. Although the disease control rate was modest (29.1%), grade 3/4 adverse events were frequently reported, including leukopenia (26%), constitutional symptoms (20%), and cutaneous disorders (14%) [11].

In Japan, IFN-β has been reported to produce a 50.0% clinical response rate in melanoma patients [12]. Based on these findings, a randomized phase III trial (JCOG1309) is currently underway to evaluate the efficacy of postoperative local administration of IFN-β in melanoma patients [2].

### 4.2. High Dose IL-2 Therapy

IL-2 is an inflammatory cytokine predominantly secreted by antigen-stimulated CD4+ T cells. It promotes lymphocyte proliferation and supports the maintenance of CD4+ regulatory T cells (Tregs) in vivo. The administration of high-dose IL-2 in combination with autologous lymphokine-activated killer (LAK) cells was first reported to induce tumor regression in an observational study involving 25 patients with metastatic cancer [13]. In that study, four out of seven advanced melanoma patients (57%) exhibited regression of metastatic lesions.

In a separate analysis of 270 melanoma patients treated with IL-2 therapy, the overall objective response rate (ORR) was 16%, consisting of a 6% complete response rate and a 10% partial response rate [14]. A retrospective analysis of 631 advanced melanoma cases treated with IL-2 alone, IL-2 combined with interferon-α, or IL-2 with chemotherapy across 12 institutions suggested that IL-2 therapy may be associated with long-term survival [3].

However, high-dose IL-2 therapy may also stimulate regulatory T cells, thereby suppressing immune responses against tumor-associated antigens. Vascular leak syndrome has been identified as a major adverse event during this therapy [15]. Due to its severe toxicity and the complexity of administration, the use of high-dose IL-2 has become limited in clinical practice [16].

## 5. Immune Checkpoint Inhibitors (ICIs) for Advanced Melanoma

As described above, although IFN-α2b and high dose IL-2 therapy showed potential in improving prognosis in earlier clinical trials, many melanoma patients were unable to continue these treatments due to severe toxicities, such as fatigue, fevers, arthralgias, liver function abnormalities, neutropenia, and nausea [17]. Additionally, high-dose IL-2 therapy was associated with serious adverse events, including hypotension secondary to capillary leak syndrome and systemic inflammatory response syndrome (SIRS)-like symptoms [18]. In light of these significant adverse events, the demand for novel therapeutic strategies led to the development of ICIs.

### 5.1. Anti-CTLA-4 Ab Monotherapy

The first T cell-attenuating receptor identified was CTLA4. It was demonstrated that the blockade of CTLA-4 by antibodies such as ipilimumab removes inhibitory signals from the co-stimulatory pathway, thereby enhancing the immune rejection of tumors in preclinical models [19]. CTLA-4 (CD152) and CD28 are homologous receptors expressed on both CD4+ and CD8+ T cells, but they mediate opposing functions. CTLA-4 binds to CD80 or CD86 on antigen-presenting cells (APCs) and inhibits T cell activation [20].

Ipilimumab was the first ICI approved by the U.S. FDA for the treatment of advanced melanoma in 2011 [7]. In a phase III trial (NCT00094653), ipilimumab at 3 mg/kg, with or without a gp100 peptide vaccine, significantly improved overall survival (OS) in previously treated advanced melanoma patients compared to the gp100 peptide vaccine alone. The 3-year OS rates were 15% for ipilimumab plus gp100, 25% for ipilimumab plus placebo, and 10% for gp100 plus placebo [21,22] (Table 2). In a long-term follow-up study comparing two ipilimumab dosing regimens, the 3-year OS rates were similar between the 3mg/kg and 10 mg/kg groups (21% [95% CI, 17–24%] vs. 24% [95% CI, 21–28%]) [23]. Overall, ipilimumab provided a better prognosis than previous chemotherapeutic agents or IL-2 therapy.

**Table 2:** First- and second-line immunotherapies for melanoma.

		Treatment	* ORR	PFS	OS	Phase	Reference
First line	Monotherapy	ipilimumab	11%		the 3-year OS: 21–25%	phase III	[22,23]
		nivolumab	42%	the 5-year PFS: 28%	the 5-year OS: 39%	phase III	[24]
		pembrolizumab	51.1%	the 7-year PFS: 23.8%	the 7-year OS: 37.8%	phase III	[25]
	Combination therapy	nivolumab + ipilimumab	58%	the 6.5-year PFS: 34%	the 6.5-year OS: 49%	phase III	[26]
		nivolumab + relatlimab	43.7%	the 36-month PFS: 31.8%	the 36-month OS: 54.6%	phase III	[27]
Second line	Combination therapy	nivolumab + ipilimumab	28%	the 6-month PFS: 34%		phase II	[28]
		nivolumab + relatlimab	9.2–12%	the 6-month PFS: 27.7–29.1%		phase I/IIa	[29]
		nivolumab + TM5614	25.7%			phase Ⅱ	[30]

* ORR: overall response rate, PFS: progress free survival, OS: overall survival.

### 5.2. Anti-PD-1Ab Monotherapy

Ipilimumab monotherapy has demonstrated tumor regression, including durable complete responses in some melanoma patients, raising expectations for immune checkpoint-targeted therapies. However, serious immune-related adverse events (irAEs) remain a concern with ipilimumab monotherapy [31]. PD-1 is another immune checkpoint expressed on T cells, B cells, natural killer T cells, activated monocytes, and dendritic cells (DCs) [32,33]. PD-1 interacts with its ligands, PD-L1 and PD-L2, to inhibit T cell proliferation, cytokine production, and cytotoxic T lymphocyte function [33,34].

In a phase I trial (NCT00730639), nivolumab was administered to 39 patients with refractory solid tumors, including melanoma, to evaluate safety, clinical efficacy, and pharmacodynamics. Objective responses were observed in melanoma patients [35]. In the CheckMate 066 trial, the nivolumab group (3 mg/kg every 2 weeks) showed better outcomes than the dacarbazine group (1000 mg/m^2^ every 3 weeks) in advanced melanoma at the 5-year endpoint (5-year OS: 39% vs. 17%; PFS: 28% vs. 3%, respectively) [36] (Table 1). In the CheckMate 067 study, nivolumab was superior to ipilimumab in melanoma-specific survival (MSS) at the 10-year endpoint (10-year MSS: 49.4 months [95% CI, 35.1 to 119.4] vs. 21.9 months [95% CI, 18.1 to 27.4]; hazard ratio [HR]: 0.59 [95% CI, 0.49 to 0.73]) [37]. A pooled analysis of 935 patients from the CheckMate 066, 067, and 069 trials showed that the 5-year OS was significantly higher in the nivolumab responder group (CR, PR) at 74% (95% CI: 72.0–84.5%) compared to 33% (95% CI: 27.5–38.0%) in the non-responder group (SD, PD) (HR: 0.26 [95% CI: 0.18–0.37]) [24]. Similarly, the 5-year PFS was significantly higher in the responder group (57% [95% CI: 48.5–64.4%]) than in the non-responder group (35% [95% CI: 26.5–44.1%]) (HR: 0.49 [95% CI: 0.35–0.69]) [24].

In the KEYNOTE-006 study, pembrolizumab (anti-PD-1 antibody) improved both OS and PFS compared to ipilimumab monotherapy (7-year OS: 37.8% vs. 25.3%; 7-year modified PFS: 23.8% vs. 13.3%) [25] (Table 1). A retrospective study found that pembrolizumab showed comparable clinical benefit to nivolumab in unresectable melanoma (3-year OS: 34% vs. 37%, respectively) [38].

Due to the underrepresentation of Asian populations in these trials, the efficacy of anti-PD-1 antibodies in these populations remains unclear. Bai et al. reported that White patients had higher objective response rates (ORR) and longer PFS compared to East Asian, Hispanic, and African patients in both non-acral cutaneous and unknown primary melanoma types [39]. Moreover, in Japanese cohorts, acral melanoma, a common subtype in East Asian, Hispanic, and African populations, responded poorly to anti-PD-1 therapy (ORR: 16.6% [CR: 3.1%; PR: 13.5%]; median OS: 18.1 months) [40].

In the CheckMate 238 trial, nivolumab was compared with ipilimumab as adjuvant therapy in resected stage IIIB–C or stage IV melanoma [41,42]. Nivolumab showed superior distant metastasis-free survival (DMFS) and OS at 5 years (5-year DMFS: 58% vs. 51%, HR = 0.79 [95% CI: 0.63–0.99]; 5-year OS: 76% vs. 72%, HR = 0.86 [95% CI: 0.66–1.12]) [42]. The EORTC1325/KEYNOTE-054 study showed that postoperative pembrolizumab significantly improved recurrence-free survival (RFS) and DMFS compared to placebo (7-year RFS: 50% vs. 36%, HR: 0.63 [95% CI: 0.53–0.74]; 7-year DMFS: 54% vs. 42%, HR: 0.64 [95% CI: 0.54–0.76]) [43,44]. As stage IIB/C melanomas have a worse prognosis than stage IIIA/B, adjuvant therapy is considered necessary [45]. In the KEYNOTE-716 trial, adjuvant pembrolizumab significantly reduced the risk of recurrence in stage IIB/IIC melanoma [46]. RFS and DMFS were both better in the pembrolizumab group than in the placebo group (36-month RFS: 76.2% vs. 63.4%, HR: 0.62 [95% CI: 0.49–0.79]; 36-month DMFS: 84.4% vs. 74.7%, HR: 0.59 [95% CI: 0.44–0.79]) [47].

**Figure 1:**
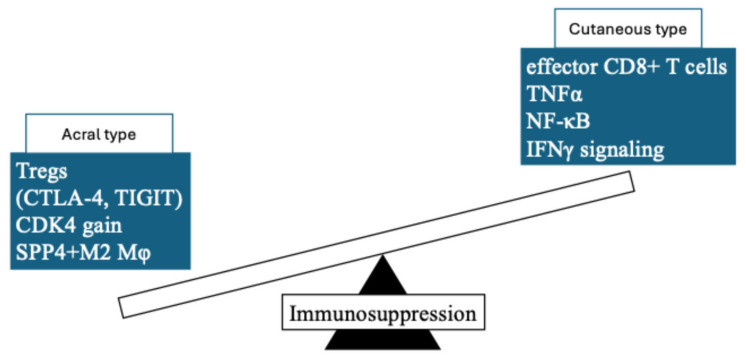
Summary of the difference in tumor microenvironment between acral and cutaneous melanoma.

Several studies have already reported potential causes for the differences in ICI treatment efficacy associated with ancestral background [48,49,50]. For example, Chinese melanoma patients in the anti-PD-1 non-responder group exhibit a significantly higher frequency of CDK4 copy number gain, especially in acral melanoma [49]. Moreover, tumors with CDK4 gain show suppressed expression of inflammation-related pathways, such as TNFα-NF-κB and IFNγ responses [49]. In another report, single-cell RNA sequencing for Japanese melanoma cohorts revealed that acral melanoma has a lower frequency of tumor-reactive CD8+ T cell clusters and an increased presence of regulatory T cells (Tregs) capable of direct tumor recognition compared to cutaneous melanoma [50]. These Treg cells exhibited high expression of immune checkpoint molecules such as CTLA-4 and TIGIT, and underwent clonal expansion within the tumor [50]. Additionally, in the tumor microenvironment (TME) of acral melanoma, macrophages displayed immunosuppressive M2-like features. In particular, SPP1 (osteopontin)-expressing macrophages were enriched in the tumor core and inhibited T cell infiltration [50]. Taken together, these studies suggest that molecular profiling-based stratification may become important for immunotherapy using ICIs, such as anti–PD-1 Ab, in the future (Figure 1).

In the CheckMate 76K trial, adjuvant nivolumab improved RFS in stage IIB/IIC melanoma (12-month RFS: 89.0% vs. 79.4%, HR: 0.42 [95% CI: 0.30–0.59]) [51]. Unfortunately, acral and mucosal melanoma types were not included in these trials. A retrospective analysis in Japanese patients showed 1-year relapse-free survival of 25.8% for acral melanoma compared to 57.1–60% for non-acral cutaneous types [52]. At 2-year follow-up (including BRAF + MEK inhibitor therapy), survival rates were 36% for acral and 42% for mucosal types, compared to 52–74% for non-acral cutaneous types [53]. Although there was no significant difference in 3-year OS between acral and non-acral types, the subtype remains an important prognostic factor [54].

Recently, neoadjuvant anti-PD-1 therapy has gained attention for its potential to improve melanoma outcomes. In a phase Ib trial (NCT02434354), patients with resectable stage III/IV melanoma received pembrolizumab (200 mg) before surgery, followed by 1 year of adjuvant therapy [55]. Long-term outcomes showed that patients achieving a complete pathologic response had favorable 5-year OS, regardless of recurrence (16.7%; 5 of 30 patients) [56]. In a phase II trial (NCT03698019), 313 patients with stage IIIB–IVC melanoma were randomized to receive neoadjuvant pembrolizumab (up to three doses) followed by adjuvant pembrolizumab (15 doses), or surgery followed by adjuvant pembrolizumab. The neoadjuvant group showed significantly better 2-year event-free survival (72% [95% CI: 64–80] vs. 49% [95% CI: 41–59], *p* = 0.004) [57]. These findings suggest that neoadjuvant or adjuvant PD-1 antibody therapy may be a promising option for achieving long-term survival in melanoma.

## 6. Combination Therapy

### 6.1. Combination with Anti-PD-1 and Anti-CTLA-4 Antibody

Although anti-PD-1 or anti-CTLA-4 monotherapy was effective in advanced melanoma, long-term outcomes were limited. In the CheckMate 067 trial, the combination of nivolumab and ipilimumab showed better clinical benefit in the 6.5-year OS rates compared to monotherapy (combination therapy: 49% vs. nivolumab: 42% vs. ipilimumab: 23%) [26] (Table 1). Due to the low proportion of Asian participants in these clinical trials, there were limitations in evaluating the efficacy in this population. In a Japanese cohort of advanced acral-type melanomas (palm and sole melanoma, *n* = 180; nail apparatus melanoma, *n* = 74), the efficacy of combination therapy was not superior to that of anti-PD-1 monotherapy for palm and sole melanoma (the ORR: 19% vs. 31%, *p* = 0.44; PFS: 5.9 vs. 3.2 months, *p* = 0.74) [58]. In a cohort of 329 Japanese patients with mucosal melanoma, there were no significant differences between the monotherapy and combination groups in ORR (26% vs. 29%; *p* = 0.26), PFS (median PFS: 5.9 vs. 6.8 months; *p* = 0.55), or OS (median OS: 20.4 vs. 20.1 months; *p* = 0.55) [59]. Although combination therapy with nivolumab plus ipilimumab improved prognosis, a major problem is the higher frequency of serious adverse events (AEs) compared to nivolumab monotherapy. Additionally, approximately 30% of melanoma patients who initially showed a clinical response were reported to subsequently develop resistance and tumor progression within 3 years of initiating ICI therapy [60].

In the SWOG S1616 trial, the combination of nivolumab and ipilimumab showed better ORR and PFS than ipilimumab monotherapy in metastatic melanoma resistant to anti-PD-1/L1 therapy (ORR: 28% [90% CI: 19–38%], 9% [2–25%]; 6-month PFS: 34% [90% CI; 25–43%] vs. 13% [4–27%]) [28]. On the other hand, there were no significant differences in PFS and OS between second-line combination therapy and ipilimumab monotherapy in 108 patients with advanced acral melanoma [61]. There remains a global need for effective second-line therapies in melanoma patients with acquired resistance.

Combination therapy with ipilimumab plus nivolumab has also been evaluated in the neoadjuvant setting in several clinical trials, similar to nivolumab monotherapy. In the OpACIN trial, 20 high-risk patients with stage III melanoma were assigned to either a neoadjuvant combination therapy group or an adjuvant combination therapy group after complete lymph node resection (doses; ipilimumab 3 mg/kg and nivolumab 1 mg/kg) [62]. Objective responses were achieved in 78% of patients treated in the neoadjuvant arm, while 90% of patients experienced severe adverse events (greater than grade 3) [62]. The OpACIN-neo trial enrolled 89 patients with stage III resectable melanoma to determine the optimal dose of neoadjuvant combination therapy [63]. Two cycles of ipilimumab 1 mg/kg plus nivolumab 3 mg/kg prior to surgery were found to be more effective and less toxic [63]. In a phase III trial, 423 patients with stage III melanoma were randomized to a neoadjuvant group (two cycles of ipilimumab plus nivolumab followed by surgery) or an adjuvant group (surgery followed by 12 cycles of adjuvant nivolumab) [64]. The estimated 12-month event-free survival was 83.7% (99.9% CI: 73.8–94.8) in the neoadjuvant group and 57.2% (99.9% CI: 5.1–72.7) in the adjuvant group [64]. Serious adverse events (Grade 3 or higher) occurred in 29.7% of the neoadjuvant group and 14.7% of the adjuvant group [64]. Since the neoadjuvant combination group showed better prognosis than the adjuvant group, it should be directly compared with anti-PD-1 monotherapy in the neoadjuvant setting.

### 6.2. Combination with Anti-PD-1 and Anti-LAG-3 Antibodies

Lymphocyte activation gene 3 (LAG-3) has been implicated in the negative regulation of effector CD8+ T cell function and major histocompatibility complex (MHC) class II activation [65]. In the RELATIVITY-047 study, relatlimab (a LAG-3 blocking antibody) combined with nivolumab was superior to nivolumab monotherapy in untreated advanced melanoma in terms of 12-month progression-free survival (PFS: 47.7% vs. 36.0%) [66]. The 3-year PFS was 31.8% (95% CI: 26.6–37.1) in the combination group and 26.9% (95% CI: 22.1–31.9) in the monotherapy group [27]. The 3-year OS was 54.6% (95% CI: 49.2–59.6) in the combination group and 48.0% (95%CI, 42.7–53.1) in the monotherapy group [27]. However, in an indirect treatment comparison, the combination of nivolumab and relatlimab did not show significant difference in OS or RFS compared to nivolumab plus ipilimumab (36-month PFS: 36% vs. 39%; 36-month OS: 48% vs. 50%, respectively) [67]. In the phase I/IIa RELATIVITY-020 trial, patients with refractory melanoma who had previously received anti-PD-1 therapy were treated with the combination of nivolumab and relatlimab [29]. The ORR was 12.0% (95% CI; 8.8–15.8) in group D1 (only one prior anti-PD-1-containing regimen) and 9.2% (95% CI; 5.2–14.7) in group D2 (multiple prior lines of anti–PD-1/L1 regimens) [29] (Table 1).

In the neoadjuvant setting, 30 patients with resectable stage III or IV melanoma were treated with two doses (nivolumab 480 mg and relatlimab 160 mg every 4 weeks) before surgery, followed by an additional ten doses (NCT02519322) [68]. The combination therapy resulted in a 57% pathological complete response (pCR) rate and 70% overall pathological response [68]. No severe adverse events were reported in this study [68].

### 6.3. Combination with Anti-PD-1 Antibody and Pegylated Interleukin-2 (IL-2) Cytokine Prodrug

Bempegaldesleukin (NKTR-214) has been developed as a novel pegylated interleukin-2 (IL-2) cytokine prodrug that activates effector CD8+ T cells and NK cells within the tumor microenvironment [69,70,71]. In the phase III PIVOT IO 001 trial, 783 patients with unresectable melanoma were treated with either bempegaldesleukin (BEMPEG) plus nivolumab or nivolumab monotherapy. The ORR was 27.7% in the BEMPEG plus nivolumab group compared to 36.0% in the nivolumab monotherapy group, showing no statistically significant difference. Similarly, the median OS was 29.67 months in the combination group versus 28.88 months in the nivolumab monotherapy group, also with no significant difference [16].

## 7. Future Perspectives

### 7.1. KIT Inhibitor Therapy Plus Pembrolizumab

The KIT gene is a proto-oncogene that encodes a transmembrane receptor tyrosine kinase. This receptor can activate the mitogen-activated protein kinase (MAPK) and PI3K/AKT signaling pathways, which are involved in tumor cell survival, proliferation, and the regulation of apoptosis [58]. KIT inhibitors are already established therapeutic options for gastrointestinal stromal tumors (GISTs) and systemic mastocytosis. Although KIT mutations are observed in only about 3% of all melanomas, they are more commonly found in acral and mucosal subtypes [72]. Overall, KIT mutations account for approximately 15% of these subtypes [8]. Due to the lower number of melanoma patients with KIT mutations enrolled in clinical trials, reports on therapeutic efficacy are limited. The single-arm phase II NICAM trial evaluated the safety and efficacy of nilotinib in patients with advanced acral and mucosal melanoma [73]. The ORR at 12 weeks was 19% (5/26 patients), with a median OS of 7.7 months. The most frequently reported adverse events were fatigue (75%), nausea (61%), and constipation (50%).

In a phase I trial (NCT02571036), ripretinib, a switch-control tyrosine kinase inhibitor targeting KIT and platelet-derived growth factor receptor A (PDGFRA), was administered to 26 patients with KIT-altered metastatic melanoma [74]. The ORR at 28 days was 23% (6/26 patients, including one complete response and five partial responses), and the median progression-free survival (PFS) was 7.3 months (95% CI: 1.9–13.6 months). Common adverse events included elevated lipase levels (50%) and alopecia (35%). An open-label, single-arm, phase I/II clinical trial (NCT04546074) is currently ongoing to evaluate the combination therapy of oral imatinib mesylate and pembrolizumab in patients with refractory KIT-mutant melanoma [75]. The results of this trial are highly anticipated. Although clinical data are still limited due to the low prevalence of KIT mutations in melanoma, KIT inhibitors may represent a promising therapeutic option for this subset of patients.

### 7.2. TM5614 Plus Nivolumab

As described above, various strategies have been investigated to enhance the efficacy of anti-PD-1 antibody therapy in patients with unresectable melanoma. Among tumor microenvironment (TME)-associated factors, plasminogen activator inhibitor-1 (PAI-1) has been shown to play a role in immune evasion [76]. Mechanistically, PAI-1 forms a complex with uPAR and LRP1, activating the JAK/STAT3 signaling cascade. This leads to upregulation of immune checkpoint molecules such as PD-L1 in both tumor cells and tumor-associated macrophages (TAMs) [76]. In murine B16F10 melanoma models, PAI-1 has been implicated in the upregulation of PD-L1 expression and the release of soluble PD-L1 (sPD-L1), resulting in reduced proliferation and activation of CD8+ effector T cells within the TME [76]. Additionally, PAI-1 contributes to the establishment of an immunosuppressive niche by increasing the intra-tumoral frequency of TAMs and regulatory T cells, thereby promoting tumor progression [76]. Based on these preclinical findings, a clinical trial (jRCT2021210029) was conducted to evaluate the therapeutic potential of combining TM5614, a selective PAI-1 inhibitor, with the anti–PD-1 antibody nivolumab in patients with advanced melanoma [30]. This phase I/II study, conducted between 2021 and 2023, demonstrated a promising objective response rate (ORR) of 25.7%, with a median progression-free survival (PFS) of 174 days (95% CI: 114.4–232.9) and a median overall survival (OS) of 273 days (95% CI: 209.7–337.0) [30]. Importantly, biomarker analyses in responding patients revealed decreases in serum IL-4 and tryptophan metabolites, suggesting that the observed therapeutic synergy may be mediated through inhibition of the immunoregulatory enzyme IL4I1 and downstream aryl hydrocarbon receptor (AhR) signaling [77]. The safety profile was favorable, with grade 3/4 immune-related adverse events occurring in only 7.9% of patients [30]. Although the immunomodulatory effects of PAI-1 are complex and other anti-tumor mechanisms may also be involved, these findings support further clinical validation. Accordingly, a randomized, double-blind, multicenter phase III trial (jRCT2021240049) was launched in February 2025 to more rigorously evaluate the efficacy and safety of this combination therapy.

## 8. Conclusions

Compared to Western populations, ICIs tend to show poorer efficacy in acral and mucosal melanoma among Asian populations, underscoring the need for more effective treatment options. Although several combination therapies (e.g., anti-PD-1 plus anti-LAG-3 antibodies, or anti-PD-1 antibody plus IL-2 cytokine prodrugs) have been explored in clinical trials, they have not yielded sufficient improvements in prognosis. Therefore, the development of novel therapies is warranted, and in the near future, molecular profiling-based stratification may become more important for immunotherapy using ICIs.

## Figures and Tables

**Table 1:** The literature review performed on PubMed.

#	Retrieval Style	No. of References
#01	“Melanoma/immunotherapy” [TIAB]	22,100
#02	“Melanoma/immune checkpoint inhibitors” [TIAB] and/or “ICIs” [TIAB])	8775
#03	“Melanoma/anti-PD-1 antibody” [TIAB] and/or “anti-PD-1 Ab” [TIAB])	2745
#04	“Melanoma/nivolumab” [TIAB] and/or “pembrolizumab” [TIAB]	14,168
#05	“Melanoma/nivolumab” [TIAB] and/or “ipilimumab” [TIAB]	7910
#06	“Melanoma/nivolumab” [TIAB] and/or “relatlimab” [TIAB]	3182
#07	“Melanoma/anti-PD-1 antibody resistant” [TIAB]	262
#08	“Melanoma/clinical trial” [TIAB] and “nivolumab” [TIAB] and/or “pembrolizumab” [TIAB]	12,495
#09	“Melanoma/clinical trial” [TIAB] and “nivolumab” [TIAB]	495
#10	“Melanoma” [TIAB] and “pembrolizumab” [TIAB]	453
#11	“Melanoma” [TIAB] and “anti-PD-1 antibody” [TIAB]	254

## Data Availability

No new data were created or analyzed in this study. Data sharing is not applicable to this article.

## Abbreviations

The following abbreviations are used in this manuscript:

MDPI	Multidisciplinary Digital Publishing Institute
DOAJ	Directory of open access journals
TLA	Three letter acronym
LD	Linear dichroism
PD-1	Programmed cell death protein 1
CTLA4	Cytotoxic T-lymphocyte antigen-4
BRAF	B-Raf proto-oncogene, serine/threonine kinase
IL-2	Interleukin-2
IFN	Interferon
NK cells	Natural killer cells
MEK	Mitogen-activated protein kinase
